# Social Distancing during COVID-19 Pandemic among Inflammatory Bowel Disease Patients

**DOI:** 10.3390/jcm10163689

**Published:** 2021-08-20

**Authors:** Michał Łodyga, Katarzyna Maciejewska, Piotr Eder, Katarzyna Waszak, Kamila Stawczyk-Eder, Agnieszka Dobrowolska, Aleksandra Kaczka, Anita Gąsiorowska, Beata Stępień-Wrochna, Małgorzata Cicha, Grażyna Rydzewska

**Affiliations:** 1 Department of Gastroenterology with the Inflammatory Bowel Disease Subdivision, Central Clinical Hospital of the Ministry of the Interior and Administration, 02-507 Warsaw, Poland; edu_kate@interia.pl (K.M.); beata.stepien.md@gmail.com (B.S.-W.); grazyna.rydzewska@cskmswia.pl (G.R.); 2 Department of Gastroenterology, Dietetics and Internal Medicine, Poznań University of Medical Sciences, Heliodor Święcicki University Hospital, 60-355 Poznań, Poland; piotreder@ump.edu.pl (P.E.); klimkaska@wp.pl (K.W.); kamilastawczyk@wp.pl (K.S.-E.); agdob@ump.edu.pl (A.D.); 3 Department of Gastroenterology, Medical University of Łódź, 92-213 Łódź, Poland; akaczka@wp.pl (A.K.); anita@sofcom.pl (A.G.); 4 Diagnostic Laboratory, Central Clinical Hospital of the Ministry of the Interior and Administration, 02-507 Warsaw, Poland; malgorzata.cicha@cskmswia.pl; 5 Collegium Medicum, Jan Kochanowski University, 25-317 Kielce, Poland

**Keywords:** SARS-CoV2, inflammatory bowel disease, social distancing

## Abstract

(1) Background: Social distancing rules have been widely introduced in the fight against the coronavirus disease 2019 (COVID-19) pandemic. So far, the effectiveness of these methods has not been assessed in the group of inflammatory bowel disease (IBD) patients. (2) Methods: The study included 473 patients with IBD who made 1180 hospital visits from 1 May to 30 September 2020. During each visit, the patients completed a five-step, progressive scale that was developed to assess the degree of social isolation. In parallel, other demographic data were collected and the concentrations of anti-severe acute respiratory coronavirus 2 (SARS-CoV-2) IgG and IgM+IgA antibodies were measured using the ELISA method. (3) Results: The study found a significant correlation between the degree of social distancing and the presence of anti-SARS-CoV-2 antibodies in the groups with the lowest degree of isolation (3 to 5). (4) Conclusions: Maintaining social distancing is an effective method for reducing the spread of SARS-CoV-2 virus among IBD patients.

## 1. Introduction

The effects of social distancing during the COVID-19 pandemic, caused by the SARS-CoV-2 virus, in patients with inflammatory bowel disease (IBD), including Crohn disease (CD) and ulcerative colitis (UC), has not been the subject of separate studies so far. However, this group requires special attention due to the chronic, often recurrent nature of the disease, as well as the very frequent and long-lasting use of immunosuppressants.

In response to COVID-19, significant efforts have been made worldwide to contain the spread of infection. The very high infectivity, even with relatively low mortality, has caused understandable concerns for the health and lives of millions of people. In the absence of effective antiviral drugs and vaccines in the early stages of a pandemic, traditional methods proven in previous epidemics have become the primary management. These include isolation of infected people, quarantine of suspected infected people, and social distancing [1].

Social distancing is the entirety of behaviors aimed at reducing contact between people, and thus reducing the risk of spreading the virus. This definition includes a wide spectrum of behaviors that can be considered at the level of entire societies (e.g., limiting movement between countries and banning mass events), smaller populations (e.g., limiting gatherings, and closing cinemas and restaurants), and individuals (e.g., remote work, and limiting social and family meetings). Previous studies are based on the assessment of social distancing at a population level, while there are few studies assessing this aspect at the level of an individual. The principles of these restrictions are largely based on the experience of fighting flu epidemics and previous coronavirus epidemics (Severe Acute Respiratory Syndrome (SARS) caused by the SARS-CoV in 2002, and Middle East Respiratory Syndrome (MERS) caused by the MERS-CoV in 2012) [2,3,4,5,6]. Although these methods have proved very effective in the SARS-CoV epidemic, the biology of the new virus (named SARS-CoV-2) has made it much more difficult to fight. First of all, this virus is characterized by a very high, up to 80%, percentage of asymptomatic cases. Asymptomatic people, in contrast with the SARS-CoV virus, can infect others in large numbers. Moreover, even people who develop symptoms (so that we can identify and isolate them) are infectious to other people for a few days before the first symptoms appear [7,8,9,10].

Due to these factors, the isolation of symptomatic cases does not prevent the spread of the virus. Similarly, quarantining people from contact with infected patients does not guarantee the containment of an epidemic. These methods may delay and mitigate the epidemic wave, as long as they are applied very early in the epidemic.

Earlier reports, including personal observations [11], suggest that IBD patients are more likely to be infected with SARS-CoV-2 than the rest of the population, although in a higher percentage present as asymptomatic [12]. For this reason, an important issue is whether adherence to the principles of social distancing in this group of patients is an effective tactic of preventing infection.

The effect of drugs used in the treatment of IBD should be considered in the context of the risk of SARS-CoV-2 infection and the risk of severe COVID-19. The data collected so far are often inconclusive. Most of them, however, do not find a significant effect of drugs, including immunosuppressants, on the increased risk of SARS-CoV-2 infection. The exception is steroids in high doses.

The aim of this study was to assess the effect of the degree of social distancing on the spread of SARS-CoV-2 virus assessed by the seroprevalence of antibodies in the population of IBD patients in Poland.

## 2. Materials and Methods

This was a multicenter, prospective observational study evaluating the effect of various levels of social distancing on the spread of the SARS-CoV-2 virus expressed by the seroprevalence of anti-SARS-CoV-2 antibodies in Polish patients with IBD. Three tertiary centers, recruiting patients from three different geographical areas in Poland—East-central (Warsaw), western (Poznan), and south-central (Lodz)—participated in the study. The study included 473 patients with inflammatory bowel disease who made 1180 outpatient visits to IBD treatment centers from 1 May to 30 September 2020. The visits were either due to the continuation of biological treatment or due to disease exacerbation.

### 2.1. Patients’ Characteristics

Each patient, upon admission to the hospital, completed a dedicated questionnaire for the presence of symptoms indicating an infectious disease of the respiratory tract in the last seven days. The questionnaire was introduced by the Ministry of Health and it was obligatory for every patient coming to every health unit in Poland in order to identify people suspected of COVID-19 infection and to refer them for further diagnostics. A five-step, progressive scale was developed to assess the degree of social isolation. Stage 1 assumes maximum isolation, and stage 5 indicates no isolation and numerous and close interpersonal contacts. The scale is presented in Table 1. Another indicator of social distancing was an interview about the travels made by patients in the past 2 weeks, and if so, the mode of transport (private or public). In addition, demographic data (gender, age, and place of residence) and treatment applied were collected. Disease activity was assessed (Crohn’s Disease Activity Index (CDAI) for CD, and partial Mayo score for UC).

### 2.2. Laboratory Analysis

Serum samples were obtained prospectively from all IBD patients at each hospital visit. The samples were immediately stored at −80 °C. The concentrations of anti-SARS-CoV-2 IgG and IgM+IgA antibodies were measured using the ELISA method, targeting viral spike (S) and nucleocapsid (N) antigens (Vircell Microbiologists^®^, Granada, Spain). All tests were performed in the Coronavirus Laboratory Diagnostic Unit of the Central Clinical Hospital of the Ministry of the Interior and Administration in Warsaw. According to the manufacturers’ recommendations, the results were considered positive if the antibody index (defined as sample optical density/cut off serum mean optical density ×10) was above 6 in the case of IgG, and above 8 in the case of IgM+IgA.

The sensitivity, given by the manufacturer, of the anti-SARC-CoV-2 IgM+IgA test was evaluated as 88% and the specificity as 99%, and for the SARS-CoV-2 IgG test, the sensitivity was 85% and the specificity was 98%.

### 2.3. Data Assessment and Statistical Analysis

After prospective collecting of all clinical data and serum samples, seroprevalence was assessed, and the results were analyzed retrospectively. All IgM+IgA positive patients had post-hoc nasopharyngeal swabs for the detection of SARS-CoV-2 infection by polymerase chain reaction (PCR).

The chi-square test of independence was used to assess the influence of the degree of social distancing and the effect of traveling on seroprevalence. The same test was also used to evaluate the effect on the degree of isolation of demographic factors, the nature of the diseases, and their treatment. Additionally, a chi-square test for trend in proportions was performed. The analysis was presented using a multifold contingency table for all values of social distance at once, and in the case of a statistically significant value, also separately for each grade (four-fold contingency table, comparing each of them with the group of others). The *p* value < 0.05 was considered statistically significant.

The Mann−Whitney U test was used for the numerical variables.

Additionally, the effect of social distancing, travel, and other factors (demographic, treatment, and disease activity) on seropositivity to SARS-CoV-2 IgG or IgM+IgA was assessed with multivariable logistic regression analysis.

The analysis was performed with the use of a Statistica 13.1 statistical package (Dell Inc., Ostin, TX, USA) and with R sofware, version 4.0.5 (R Foundation for Statistical Computing, Vienna, Austria).

### 2.4. Ethics Approval

This study was approved by the Ethics and Supervision Committee for Human and Animal Research at the Central Clinical Hospital of the Ministry of the Interior and Administration in Warsaw (no. 66/2020) and the Bioethics Committee at the Poznan University of Medical Sciences (no. 364/20). All of the patients provided written informed consent to participate in the study.

## 3. Results

First, 473 subjects were enrolled in the study: 319 with CD and 154 with UC. During the study period, these patients had a total of 1180 visits (one to seven visits per patient, mean 2.2).

The associations between the level of social distancing and seropositivity against SARS-CoV-2 IgG or IgM+IgA and the demographical and clinical variables are shown in Table 2 and Table 3.

The associations between traveling and seropositivity against SARS-CoV-2 IgG or IgM+IgA are shown in Table 4.

The *p* value at the end of each row is presented for all values of social distancing at once, and in the case of a statistically significant value, also separately for each grade (comparing each of them with the group of others).

The mean age for the entire group was 35.6 years, with a BMI of 24.1 kg/m^2^. The mean CD activity, expressed as CDAI, was 150 and the mean Mayo score for UC was 2.94.

The study found a significant impact between the degree of social distancing and the presence of anti-SARS-CoV-2 antibodies in both the IgM+IgA and IgG class (using chi-square tests for trends in proportions). The relationship was expressed by a higher percentage of positive antibody results in the groups with the lowest degree of isolation (for IgM+IgA, 21.63% of positive results in group 3, 33.93% in group 4, and 41.82 in group 5, *p* < 0.001, and for IgG, 18.18% of positive results in group 5, *p* = 0.0204).

In the analysis of the influence of other factors on compliance with the degree of social isolation, it was found that among CD patients, there was a greater percentage of people who did not completely undergo social isolation (group 5, 14.15% versus 5.23% among UC patients, *p* = 0.0045).

In the case of Crohn’s disease, a significant influence of the disease activity (expressed using the CDAI scale) was found. Among the patients who do not adhere to the principles of social distancing, the average CDAI was the lowest (CDAI 106 in group 5 compared with CDAI 150 in whole population). It should be noted, however, that the observed mean CDAI values were similar to the values considered to be clinical remission (150) in all groups.

Analyzing the effect of the treatment used, a significantly higher level of social isolation was found in patients using steroids. The effect of other drugs, including immunosuppressants and biological drugs, has not been proven.

There was also a higher proportion of patients with a positive SARS CoV 2 IgM+IgA antibody result among the patients who declared traveling outside the place of residence in the last 2 months (32.52% compared with 23.26 in the whole population, *p* = 0.0047). The type of mode of transport (private or public) had no effect on the result.

No significant correlation was found between the degree of social isolation and gender, age, or number of inhabitants in the place of residence.

No symptomatic COVID-19 was found in any of the patients during the entire follow-up period.

Additionally, the influence of social distancing, traveling, and other factors (demographic, treatment, and disease activity) on seropositivity against SARS-CoV-2 IgG or IgM+IgA was evaluated using a multivariable logistic regression analysis. The analysis confirmed the influence of social distancing and travel on the presence of antibodies (Table 5). In the case of travel, this concerned the impact of travel by public transport on the increased risk of the presence of IgA+IgM antibodies. BMI was an additional factor that modified the presence of IgM+IgA antibodies. No other demographic factor, treatment, or disease activity influenced the presence of the antibodies.

## 4. Discussion

In order to assess the degree of social distancing, a scale of social isolation used during the study was developed. So far, from what we know, there has been no good tool to evaluate it at the level of individual behavior. While the issue of social distancing has been the subject of intense research during the current pandemic, it has been focused on population research [1,2,10,13,14,15]. These have proven the effectiveness of social distancing at reducing the spread of the virus. The impact of limiting interpersonal contact in a given area (expressed with various variables) on reducing the number of infections was examined. No universally accepted scale has been developed anywhere to assess the degree of isolation of an individual. Therefore, the proposed scale was created for the purpose of this study.

Most studies available to date are based on the assessment of symptomatic COVID-19 cases [13,14,15]. Only a few have looked at asymptomatic cases, the numbers of which are very high for the SARS-CoV-2 virus and critical to the maintenance of the pandemic [11,12,16,17,18]. The present study focuses on the assessment of asymptomatic cases or contact of patients with an infected person, expressed by antibody seroprevalence. Of course, the serological diagnosis of SARS-CoV-2 infection is not a precise method to assess the existence of asymptomatic infection in a particular patient, but it is a sufficient tool for the evaluation of virus penetration in a given population [19]. Testing the presence of antibodies in large populations is difficult and costly, which is why it is so important to collect data for smaller, specific groups, such as in the present study.

At the time the study was conducted, Poland was a country moderately affected by the pandemic. Initially, restrictions on the recommendation of online work; restrictions on the operation of cinemas, restaurants, and gatherings; limiting the number of people in public transport; online learning in schools; and the requirement to wear masks in public spaces were gradually reduced in June 2020. However, it was recommended to increase social distancing and wear masks in confined spaces. This study proves that the number of asymptomatic infections is increasing among patients who have not adhered to the principles of social distancing. However, it has not been shown that in the group of patients who adhere to these rules the percentage of people with positive antibodies was lower. This may be due to the small group of patients who most closely followed the rules of isolation. The reason for this is the specificity of the studied group. They were people with IBD, mostly biologically treated. These patients had to visit the treatment center for drug administration, follow-up visits, or due to disease exacerbation. Nevertheless, the presented data are sufficient to confirm the effectiveness of social isolation in reducing the spread of SARS-COV-2 virus in the study group.

The presented study shows a significant impact of patient education on the observance of the principles of social distancing. People using steroids—drugs potentially increasing the risk of severe infection and patients with CD—were more prone to isolation. On the other hand, patients who did not use any social isolation were characterized by the lowest disease activity. Therefore, physicians should recommend that patients with IBD adhere to the principles of social distancing, regardless of disease activity or medications, especially as IBD mainly affects young people who are less willing to submit to administrative restrictions. In addition, the long duration of the pandemic reduces the tendency to comply with strict isolation rules over time [20].

The present study does not take into account other non-pharmacological measures to reduce the spread of the virus. One of the most common is the use of masks [21,22]. In line with the regulations enforced at the time of the study, all patients and healthcare professionals wore masks at all times during their visits to the center.

Can the presented results be related to the general population as evidence of the effectiveness of isolation rules in the fight against the COIVD-19 pandemic? They are consistent with the available research; however, both the methodology and the study group are innovative. Undoubtedly, these results are another argument in favor of applying the principles of isolation.

After many months of fighting the pandemic, opposition to the imperative of social isolation is growing in many societies [20,23]. However, the results of studies such as those presented here indicate the correctness of the strategy of increasing social distance as an element of mitigating the wave of the pandemic. While the mass vaccination program offers hope to end the pandemic’s nightmare and isolation orders are being relaxed, the emergence of more and more variants of the virus makes the future uncertain [24,25,26,27,28]. Perhaps it will be necessary to return to social isolation, and this research will be one of the arguments in favor of its introduction.

The presented work has some limitations. In the study population, there were relatively few people who adhered to the principles of social distancing the most (grade 1—complete social isolation). This is due to the study population in which there were people who had to go to an IBD treatment center for continuation of treatment. The proposed scale of social distancing is a new tool and has not been studied on a larger population so far. The results of the study may also affect other methods used to reduce the spread of the virus, such as the obligation to use masks. In addition, a study through questionnaire is exposed to the risk of misunderstanding and bias. 

## 5. Conclusions

The scale of the degree of isolation presented in the study can be a useful tool for assessing the effectiveness of actions taken to reduce the spread of COVID-19, as well as other infections in the future, but undoubtedly it requires further research.

Together with the authors’ earlier research, this study suggests a large effort that should be placed on education and convincing patients with IBD to apply social distance principles, regardless of the severity of the disease and medicines used.

Furthermore, a great effort should be made to reduce the risk of health-care related infection by changing the rules of organization of the centers.

## Figures and Tables

**Table 1:** The proposed scale of the degree of social isolation.

Social Distance Level	
1	Full isolation: I live alone, I do not leave the house
2	I live alone, work remotely, I leave the house only when necessary, keeping a distance of 2 meters from other people
3	I live with my family, I work remotely, I leave the house several times a day keeping a distance of 2 meters from other people, I do not use public transport
4	I live with my family, go to work/use public transport
5	No isolation: I meet a lot of people every day

**Table 2:** The associations between level of social distancing and seropositivity against SARS-CoV-2 IgG or IgM+IgA.

Social Distance Level	1	2	3	4	5	*p* Value
*n* (%)	13 (2.75)	48 (10.15)	245 (51.80)	112 (23.68)	55 (11.63)	
Positive anti SARS-CoV-2 IgM+IgA results, *n* (%), *p*	1 (7.69), 0.1278	8 (16.67), 0.1217	53 (21.63), 0.0266	38 (33.93), 0.0329	23 (41.82), 0.0043	*p* < 0.0001 *
Positive anti SARS-CoV-2 IgG results, *n* (%), *p*	1 (7.69), 0.8412	2 (4.17) 0.1976	20 (8.16), 0.3850	11 (9.82), 0.8496	10 (18.18), 0.0156	*p* = 0.0204 *

* Chi-square test for trend in proportions.

**Table 3:** The associations between level of social distancing and demographical and clinical variables.

Social Distance Level	1	2	3	4	5	*p* Value
*n* (%)	13 (2.75)	48 (10.15)	245 (51.80)	112 (23.68)	55 (11.63)	
Demographic						
Sex, *n* (%) female	5 (2.56)	18 (9.23)	111 (56.92)	46 (23.59)	15 (7.69)	0.1699
male	8 (2.88)	30 (10.79)	134 (48.20)	66 (23.74)	40 (14.39)	
Age, y, SD	33.0 ± 16.98	37.9 ± 16.3	35.0 ± 12.57	37.1 ± 11.08	34.1 ± 9.01	0.1282
BMI, kg/m^2^, SD, *p*	22.6 ± 3.94, 0.3366	24.2 ± 4.79, 0.7663	24.0 ± 5.25, 0.0765	24.2 ± 4.58, 0.7121	25.7 ± 4.72, 0.0081	0.0074
Place of residence						
<1000 inhabitants, *n* (%)	1 (1.22)	8 (9.76)	43 (52.44)	18 (21.95)	12 (14.63)	0.1509
1000–10,000 inhabitants, *n* (%)	5 (9.62)	3 (5.77)	28 (53.85)	9 (17.31)	7 (13.46)	
10,000–100,000 inhabitants, *n* (%)	3 (2.50)	9 (7.50)	66 (55.00)	28 (23.33)	14 (11.67))	
>100,000 inhabitants, *n* (%)	4 (1.83)	28 (12.79)	108 (49.32)	57 (26.03)	22 (10.05)	
**Treatment**						
5-ASA, *n* (%)	11 (2.72)	39 (9.65)	208 (51.49)	98 (24.26)	48 (11.88)	0.8663
Thiopurines, *n* (%)	5 (1.95)	24 (9.38)	132 (51.56)	66 (25.78)	29 (11.33)	0.6086
Anti-TNF, *n* (%)	5 (2.08)	24 (10.0)	124 (51.67)	56(23.33)	31 (12.92)	0.8237
Vedolizumab, *n* (%)	3 (2.54)	11 (9.32)	60 (50.85)	31 (26.27)	13 (11.02)	0.9569
Ustekinumab, *n* (%)	1 (6.67)	2 (13.33)	7 (46.67)	3 (20.00)	2 (13.33)	0.8738
Steroids, *n* (%), *p*	3 (3.23), 0.7503	11 (11.83), 0.5441	59 (63.44), 0.0114	16 (17.20), 0.0939	4 (4.30), 0.0141	0.0284
**Disease Activity**						
Crohn, *n* (%), *p*	10 (76.92), 0.4721	31 (64.58), 0.6229	155 (63.52), 0.0430	77 (68.75), 0.7349	45 (84.91), 0.0045	0.0423
Ulcerative colitis, *n* (%)	3 (23.08), 0.4627	17 (35.42), 0.6471	89 (36.48), 0.0552	36 (32.14), 0.8706	8 (15.09), 0.0041	0.0442
CDAI mean, *p*	129, 0.6544	167, 0.3782	159, 0.0793	156, 0.5376	106, 0.0004	0.0087
Mayo, mean, *p*	2.33, 0.8283	2.94, 0.5943	3.26, 0.0405	2.17, 0.0623	3.13, 0.9379	0.5212

SD—Standard deviation.

**Table 4:** The associations between traveling and seropositivity against SARS-CoV-2 IgG or IgM+IgA.

Travel	Yes	No	*p* Value
*n* (%)	110 (23.26)	363 (76.74)	
Positive anti SARS-CoV-2 IgM+IgA results, *n* (%)	40 (32.52)	83(67.48)	0.0047
individual transport	28 (33.33)	56 (66.67)	0.1246
public transport	13 (50.00)	13 (50.00)	
Positive anti SARS-CoV-2 IgG results, *n* (%)	12 (27.27)	32 (72.73)	0.5078

**Table 5:** Multivariate logistic regression models for seropositivity against SARS-CoV-2 IgM+IgA and IgG.

	Seropositivity against SARS-CoV-2 IgM+IgA			Seropositivity against SARS-CoV-2 IgG		
	OR	95% CI for OR	*p*	OR	95% CI for OR	*p*
Social distance level (1 = baseline)						
2	1.92	0.21 to 17.75	0.563	0.57	0.04 to 6.83	0.655
3	3.1	0.39 to 24.62	0.284	1.15	0.14 to 9.33	0.897
4	6.15	0.76 to 49.63	0.088	1.37	0.16 to 11.70	0.771
5	8.59	1.02 to 72.58	0.048	2.80	0.31 to 24.79	0.355
Travels (No travels = baseline)						
Travels—Individual transport	1.33	0.75 to 2.38	0.329	1.20	0.53 to 2.73	0.666
Travels—Public transport	2.58	1.1 to 6.05	0.029	1.14	0.32 to 4.11	0.843
BMI	0.94	0.89 to 0.99	0.014	0.97	0.90 to 1.04	0.371

OR—odds ratio with 95% confidence interval (CI).

## Data Availability

The data presented in this study are available upon request from Michal Lodyga. The data are not publicly available due to privacy restrictions.
